# Multimodal human–computer interaction in interventional radiology and surgery: a systematic literature review

**DOI:** 10.1007/s11548-024-03263-3

**Published:** 2024-10-28

**Authors:** Josefine Schreiter, Florian Heinrich, Benjamin Hatscher, Danny Schott, Christian Hansen

**Affiliations:** 1 Faculty of Computer Science and Research Campus STIMULATE, University of Magdeburg, Magdeburg, Germany; 2 Siemens Healthineers, Forchheim, Germany

**Keywords:** Human–computer interaction, Multimodal interaction, Computer-assisted medicine, Touchless interaction

## Abstract

**Purpose:**

As technology advances, more research dedicated to medical interactive systems emphasizes the integration of touchless and multimodal interaction (MMI). Particularly in surgical and interventional settings, this approach is advantageous because it maintains sterility and promotes a natural interaction. Past reviews have focused on investigating MMI in terms of technology and interaction with robots. However, none has put particular emphasis on analyzing these kind of interactions for surgical and interventional scenarios.

**Methods:**

Two databases were included in the query to search for relevant publications within the past 10 years. After identification, two screening steps followed which included eligibility criteria. A forward/backward search was added to identify more relevant publications. The analysis incorporated the clustering of references in terms of addressed medical field, input and output modalities, and challenges regarding the development and evaluation.

**Results:**

A sample of 31 references was obtained (16 journal articles, 15 conference papers). MMI was predominantly developed for laparoscopy and radiology and interaction with image viewers. The majority implemented two input modalities, with voice-hand interaction being the most common combination—voice for discrete and hand for continuous navigation tasks. The application of gaze, body, and facial control is minimal, primarily because of ergonomic concerns. Feedback was included in 81% publications, of which visual cues were most often applied.

**Conclusion:**

This work systematically reviews MMI for surgical and interventional scenarios over the past decade. In future research endeavors, we propose an enhanced focus on conducting in-depth analyses of the considered use cases and the application of standardized evaluation methods. Moreover, insights from various sectors, including but not limited to the gaming sector, should be exploited.

## Introduction

Interaction with computer interfaces is of utmost importance in medicine for various reasons, e.g., to access information during interventions or to document findings and reports. However, a sterile environment does not always permit the usage of conventional input devices such as a keyboard. Furthermore, various tasks like handling medical instruments occupy the hands. Thus, touchless approaches for human–computer interfaces (HCI) for medical use cases have been investigated for some years and reviewed thoroughly [1, 2]. Since Bolt’s *put that there* demo [3], the combination of several input modalities has offered the promise of natural interaction. Using input modalities as alternatives on the other hand provides flexibility, in case one channel is blocked with other tasks. Moreover, with multimodal (MM) interfaces, the output may also not be limited to a single sensory modality, but may make use of various visual, auditory, and haptic channels.

Researchers have investigated MM interaction (MMI) in various fields of application. They focused on the analysis of interaction technology types with regard to domains [4] and the investigation of MM human–robot interaction with specific focus on signal input and output [5]. However, no one has analyzed MM systems for surgical and radiological scenarios with particular regard to input and output modalities as well as the addressed medical tasks.

This work aims to draw a comprehensive picture of current approaches in MMI of these use cases over the past decade. A systematic literature review was performed, and the results were clustered to contribute an overview and to provide guidance for future research. In detail, we aimed to answer the following questions:

- RQ1 - How did the inclusion of input modalities change over time?
- RQ2 - For which medical fields and tasks were MMI developed?
- RQ3 - Which input modalities are provided for which tasks?
- RQ4 - What are common modalities used for system output compared to input?
- RQ5 - Which challenges are identified?

## Materials and methods

Methodologically, this review is oriented on the *Preferred Reporting Items for Systematic Reviews and Meta-Analyses* (PRISMA) [6] and the standards described in Atkinson et al. [7]. In the following, the literature review process including the search strategy, screening protocol, and eligibility criteria used to identify relevant articles is described.

**Fig. 1:**
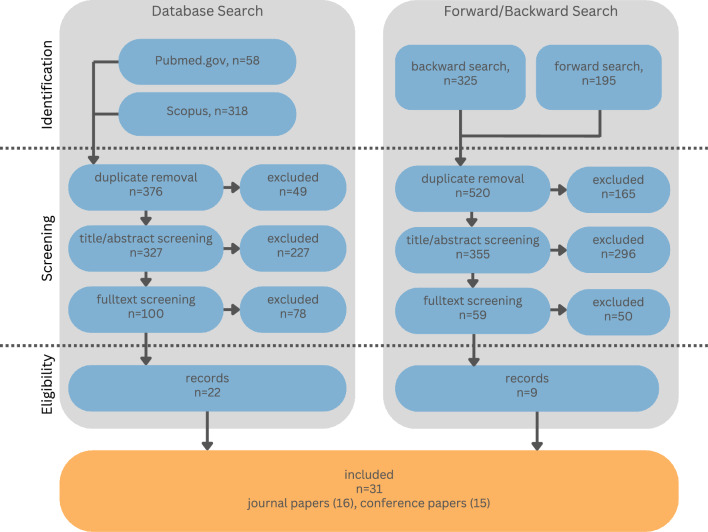
Steps and respective publication counting of the review methodology according to Moher et al. [6]

### Identification

The identification step differed between two phases. Within the first phase, a database query was conducted. The second phase applied a forward/backward search for relevant papers identified in the first iteration. Each of the applied identification methods is described below.

#### Database search

Databases were selected to gather all relevant literature within the scope of this review. Two databases were chosen to identify publications within the medical domain (PubMed.gov) as well as in a broader field of research (Scopus). The search term was constructed systematically guided by the following two steps:

1. connect topic (*multimodal interaction*) and context (*medical scenario*) with AND.
2. find synonyms or closely related terms for both general terms and connect them with OR.

The resulting search term^1^ was entered into the mentioned databases to search within title, abstract and keywords of references. Additionally, filters were applied to specify the search in terms of document type (*Conference Paper and Journal Articles*), language (*English*), and publication dates (*2013-present*).

#### Forward/backward search

In the second phase, a backward (reference list) and a forward (citation) search was performed, based on publications identified in the first iteration. The forward and backward search was conducted using *Scopus*. A database filter to limit obtained references in terms of publication date (starting from 2013) was applied.

### Screening

After identification, duplicates were removed semi-automatically. Then, titles and abstracts were screened regarding the topics (1) *multimodal human–computer interaction* (minimum of two input modalities) and (2) the *medical context* covering domains of applied surgery and interventions as well as research which could potentially be applied the preparation of or during these use cases. (Excluded were publications concerning rehabilitation and emergency medicine.) When both topics were present, the reference was included for full text screening. During the screening, records were assessed for eligibility and considered relevant if the following inclusion criteria were met and none of the exclusion criteria were present.

**Inclusion criteria**

- peer-reviewed
- open access
- at least two input modalities used for HCI^2^
- HCI technique(s) described and implemented
- clinical task, scenario, or use case (as described above)
- evaluation of the system or interaction

**Exclusion criteria**

- no contribution (e.g., Front Matter, Keynote)
- mere virtual replication of a clinical setting^3^

Both screening steps were performed by two investigators. A liberal integration approach was applied regarding the title–abstract screening: References which were considered relevant by one of the investigators were included for the next step. For full text assessment, one investigator analyzed the research articles, while articles marked as borderline cases were evaluated in collaboration with the other investigator until consensus was reached.

## Results

An overview of the literature review process as well as the respective numbers of records for each step can be found in Fig. 1. The database search was initially conducted in September 2023. Between November and December 2023, the forward/backward search followed. To guarantee the inclusion of publications until the end of 2023, the database search was performed again in January 2024. Numbers of unique records within the database search were updated, and the respective references were included in the following screening steps. The final sample comprised 31 publications, including 16 journal articles and 15 conference papers. In the following, the obtained sample is analyzed to answer the research questions stated in Sect. "Introduction".

**Fig. 2:**
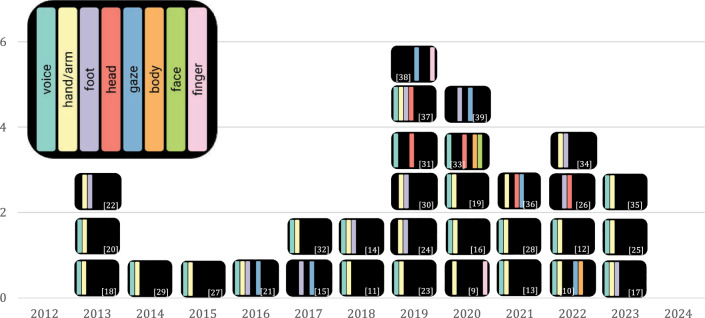
Number of publications per year, including applied input modalities. Colored bars within the rectangle indicate the type of input modality. White numbers in brackets refer to the referenced publication

### Input modalities (RQ1)

Figure 2 indicates the distribution of references within the defined time period and applied input modalities per reference. System input is often categorized according to sensory modes through which the user conveys information to the system and thus interacts with it, e.g., based on touch or vision [8]. However, we chose a classification according to distinct capabilities and characteristics of body parts or sensory channels. HCI performed by hands and arms was summarized as one category and is hereinafter referred to as *hand interaction*.

The majority of publications implemented two input modalities (24 references, 77.4%), followed by four (4 references, 12.9%) and three ways (3 references, 9.7%) to interact with the system. Nineteen references (61.3%) included a combined voice–hand interaction (of which 14 exclusively and 5 also applied other input modalities). Foot interaction was incorporated in eleven developed systems (35.5%), mostly together with hand interaction, occurring in 73% of these cases. Interactions facilitated by fingers, body, and face were applied the least in two (6.5%) and one (3.2%) of the analyzed works, respectively.

### Application (RQ2)

Table 1 shows the analysis of obtained publications in terms of the targeted medical field and tasks to be realized by the developed MMI. The frequencies of addressed fields are in descending order: 19.4% laparoscopy (6 references), 16.1% radiology (5 references), 9.7% general surgery (3 references), 6.5% neurosurgery (2 references), 3.2% each for vascular surgery, dentistry, and diagnostics (each 1 reference). 38.7% (12 references) did not specify the targeted medical field. Besides that, 94% of the systems that have been implemented (29 references) are dedicated to the fulfillment of practical medical tasks and 3% relate to training scenarios, whereas 3% did not specify the intent of use (each 1 reference).

The included references were categorized related to tasks. Twenty publications (64.5%) used their systems to handle medical *2D image data*, and 11 (35.5%) included interaction with medical *3D data*. Seven (22.6%) and three references (9.7%) referred to the manipulation of *robots* and the interaction with a custom *graphical user interfaces (GUI)*, respectively. Four publications (12.9%) found in this review implemented systems to interact with *functions of an OR* and to support *team communication*, two per category (6.5%).

The categories were further analyzed for their specific task(s) to be performed with the help of the developed MMI. Thereby, *2D navigation* includes, e.g., the selection, moving, zooming, browsing, and parameter modification of 2D images, whereas the category *3D manipulation* defines these kind of actions with 3D objects. *Robot control* includes the interaction with either an endoscope or a robotic arm. *GUI* control comprises the interaction with elements of a designated interface which is not part of a conventional system. Publications which involved systems to interact with *functions of an operating room (OR)* used it to modify the OR light and general settings which were not further specified. In two references (6.5%), the implemented MMI addresses team communication.

**Table 1:** Final sample of references included in the review with respective addressed medical field, interaction purpose, task category, and categorized task specification

References	Medical field	Purpose	Task category	Task specification
[9]	Laparoscopy	P	Robot	Endoscope navigation
[10]	N/S	P	2D image viewer, GUI	2D navigation, GUI element interaction
[11]	Laparoscopy	T	2D image viewer, team communication	support team communication, add notations/drawings
[12]	N/S	N/S	2D+3D image viewer	2D navigation, 3D manipulation
[13]	N/S	P	2D image viewer	2D navigation
[14]	N/S	P	2D image viewer	2D navigation
[15]	Radiology	P	2D+3D image viewer	2D navigation, parameter modification, 3D manipulation
[16]	N/S	P	GUI, OR functions	GUI element interaction, adjust OR light
[17]	N/S	P	3D Image viewer	3D manipulation
[18]	Radiology	P	2D Image viewer	2D navigation
[19]	Neurosurgery	P	2D+3D image viewer	2D navigation, 3D manipulation
[20]	General Surgery	P	Robot	Request robot movement
[21]	General Surgery	P	Robot	Request robot movement
[22]	N/S	P	2D image viewer	2D navigation
[23]	N/S	P	2D+3D image viewer	2D navigation, 3D manipulation
[24]	General Surgery	P	3D image viewer	3D manipulation
[25]	N/S	P	Robot	Robot navigation
[26]	Laparoscopy	P	Robot	Endoscope navigation
[27]	N/S	P	2D image viewer	2D navigation
[28]	Neurosurgery	P	2D+3D image viewer	2D navigation
[29]	Vascular Surgery	P	2D+3D image viewer	2D navigation
[30]	Dentistry	P	2D+3D image viewer	2D navigation, 3D manipulation
[31]	N/S	P	3D image viewer	3D manipulation
[32]	Laparoscopy	P	2D+3D image viewer, OR functions	2D navigation, 3D manipulation, handle OR functions (N/S)
[33]	Radiology	P	2D image viewer	2D navigation
[34]	Radiology	P	2D image viewer, GUI	Parameter modification, GUI element interaction
[35]	Radiology	P	Robot	Robot navigation
[36]	Laparoscopy	P	Robot	Endoscope navigation
[37]	N/S	P	2D image viewer	2D navigation
[38]	Diagnostics	P	2D image viewer	2D navigation
[39]	Laparoscopy	P	2D image viewer, team communication	2D navigation, support team communication

*P* Practice, *T* Training, *N/S* not specified

### Assignment of input modalities to tasks (RQ3)

An overview of assigned input modalities to tasks can be seen in Table 2. Twenty-five publications (80.6%) distinguished between discrete and continuous control. Voice input was most commonly used for the former (16 references, 51.6%) followed by foot control (9 references, 29.0%), whereas hand interaction for the latter (18 references, 58.1%), also followed by foot input (6 references, 19.4%). In contrast, four publications (12.9%) [9, 20, 21, 27] enabled the usage of all implemented input modalities to achieve all system functionalities.

Frequent discrete commands in controlling image viewers are system de/activation, mode changes, and switching the interaction type, whereas continuous control comprises navigation and manipulation of images. In systems which include robots or endoscopes, discrete commands are used for moving the robot to a predefined position in space or switching the navigation mode and continuous control to manipulate the end effector. The control of GUIs comprises the activation of specific interface elements for discrete control and continuously manipulating them (e.g., slider); like it is the case in a work of Heibeyn et al. [16]. This work also includes the control of OR functionalities, with voice being applied to call certain devices and hand control for their manipulation.

**Table 2:** Input modalities and their assignment to tasks for the control of image viewers^1^, GUIs^2^, robots^3^, OR functions^4^, and to support team communication^5^. *Abbreviations: vc-voice, ha-hand, ft-foot, hd-head, gz-gaze, bd-body, fc-face, fg-finger*

References	Input modalities								Assignment details
	vc	ha	ft	hd	gz	bd	fc	fg	
[9]^3^									All for continuous
[10]^1,2^									All for discrete
[11]^1,5^									vc for discrete, ha for continuous
[12]^1^									vc for discrete, ha for continuous
[13]^1^									All for discrete and continuous
[14]^1^									vc+hd+ft for discrete, ha+ft for continuous
[15]^1^									gz+ft for discrete, ft for continuous
[16]^2,4^									vc for discrete, ha for continuous
[17]^1^									vc+ft for discrete, ha for continuous
[18]^1^									All for continuous
[19]^1^									All for discrete and continuous
[20]^3^									All for discrete
[21]^3^									All for discrete
[22]^1^									ft for discrete, ha for continuous
[23]^1^									vc for discrete, ha for continuous
[24]^1^									ft for discrete, all for continuous
[25]^3^									vc for discrete, ha for continuous
[26]^3^									ft for discrete, hd for continuous
[27]^1^									All for discrete and continuous
[28]^1^									vc for discrete, ha for continuous
[29]^1^									vc for discrete, ha for continuous
[30]^1^									ft for discrete, ha for continuous
[31]^1^									vc for discrete, hd for continuous
[32]^1,4^									vc N/S, ha for continuous
[33]^1^									vc+hd for discrete, hd+bd+fc for continuous
[34]^1,2^									All for discrete and continuous
[35]^3^									vc for discrete, ha for continuous
[36]^3^									ha for discrete, hd+gz for continuous
[37]^1^									vc+hd+ft for discrete, ha+hd+ft for continuous
[38]^1^									All for discrete and continuous
[39]^1,5^									gz for discrete, ft for continuous

### Output modalities (RQ4)

Twenty-five references (80.6%) included system output of which two applied a MM approach (6.5%), and both of them combined visual feedback with audio cues [28, 36]. Of the 23 other publications, 22 implemented a visual output (71.0%), whereas 1 reference (3.2%) applied audio cues [17]. The audio feedback was implemented in a way that it indicates successfully recognized gestures in Heinrich et al. [17] and the switching of modes in Nishihori et al. [28] and Sivananthan et al. [36]. References that contained a visual output gave the user feedback about, e.g., an in/active interaction modality or the de/activation of certain system statuses. The feedback was provided using visual elements via augmented reality [31, 35, 37], virtual reality [12, 23, 25, 34], laparoscopic video stream [9, 11, 36], included hardware [35], projection [24], and/or external displays [10, 14–16, 19, 22, 24, 27–30, 33, 38, 39]. The visual elements were provided in the form of indicators like icons and arrows, cursors, colored display frames, dedicated GUIs, status bars, and lists with interaction commands.

### Challenges (RQ5)

To explore the challenges encountered during the development and evaluation of MM systems, the identified issues were clustered into categories, as outlined below. Regarding the development, some researchers experienced challenges in integrating the interaction into the medical environment. These included, e.g., low input recognition for reasons of medical clothing and/or a noisy surrounding [10, 27, 28]. Moreover, in several publications, study participants criticized the missing haptic feedback of touchless interaction methods. This issue particularly occurred when using medical hardware reliant on haptic feedback, such as ultrasound examination [35] and endoscope handling [9, 36]. Unergonomic body postures or movements caused by the interaction posed a further difficulty. These were especially prevalent during gaze interaction [15, 38], due to the necessity of blinking, which might inadvertently trigger unintended activation and contribute to interaction fatigue, as well as during foot interaction in a standing posture, which led to reported balance problems [22, 33].

A frequent limitation in evaluating MMI was the number of study participants. Of the 29 references (93.5%) that included a user study (excluding Gao et al. and Gulmez et al. [12, 13]), only three involved at least 20 participants [10, 17, 31], and five included at least 15 participants in the evaluation process [14, 30, 33, 34, 36]. Fourteen systems (45.2%) were evaluated by study participants who represented the intended target group [9–11, 16, 18, 19, 26–30, 32, 36, 39], and eight systems (25.8%) were evaluated involving medical students, either partially or entirely [14, 15, 17, 23, 31, 33–35]. Moreover, only seven publications (22.6%) comprised a realistic test scenario, either for training purposes [11] or for clinical practice [27–30, 32, 39]. In 14 publications (45.2%), an evaluation with standardized questionnaires was performed, of which ten used the *NASA Task Load Index* (TLX [40]) [10, 15–17, 31, 33, 34, 37–39], three the *System Usability Scale* (SUS [41]) [25, 35, 39], one reference each the *meCUE* [42] (used by Hatscher et al. [14]), and a questionnaire according to Van der Laan et al. [43] (used by Sivananthan et al. [36]). In contrast, nine MM interfaces (29.0%) were assessed using non-standardized custom questionnaires [9, 11, 18, 19, 22–24, 26, 32]. Four publications (12.9%) compared their developed MM approach with a conventional system (e.g., touch interaction for endoscope control) [11, 12, 23, 36] and five (16.1%) with an unimodal approach (one input modality of the MMI) [14, 15, 20, 30, 32].

## Discussion

In response to *RQ1*, it is noteworthy that the predominant number of publications involves interaction with voice, hand, and/or foot, while avoiding integrating input modalities such as face, body, and gaze. This tendency might be attributed to concerns about ergonomics and the dual role of certain input methods, as is the case with gaze interaction. Another presumed reason for this preference is the technical feasibility of tracking hand and voice interaction. Additionally, these modalities are considered more natural and precise compared to alternatives such as eyebrow interaction [33].

Concerning *RQ2*, the analysis revealed that laparoscopy and radiology are frequently addressed medical fields in which MMI supports in completing physically demanding actions and secondary tasks that require multitasking. This involves the handling of endoscopes and ultrasound probes, the manipulation of medical images, and the adjustment of OR settings, which often demand team interaction. Thus, MMI holds significant potential in relieving workforce strain, assisting with tasks when hands are occupied, and enabling experienced-based physically demanding actions.

In relation to *RQ3*, the prevalence of two interaction modalities in most publications may be attributed to the increased complexity of integrating more controls, alongside the presumption that adding modalities does not necessarily enhance usability. In this context, it is crucial to carefully evaluate the impact of MMI on team dynamics and work practices, as it may affect the restructuring of the work of surgeons and their teams [44, 45]. Additionally, it is noteworthy that in most systems, the assignment of specific interaction modalities to individual tasks of the medical procedure remains fixed, limiting user flexibility in selecting from various input control for a given task. The advantages and disadvantages of fixed and flexible assignment approaches should be carefully considered. Ultimately, the number of integrated input modalities and their assignment to tasks depend on individual requirements and goals of the application, as well as the available resources and technological constraints.

In the context of *RQ4*, the analysis revealed that the inclusion of visual feedback for interaction is prevalent in most references, which is not unexpected given the involvement of displays in the interaction process. However, the primary emphasis in many research is directed toward the design and implementation of input modalities. Nevertheless, the significance of system output is paramount, as it plays an important role in providing feedback, conveying information, and improving the overall user experience. It is suggested that the significance and impact of MM feedback should be investigated in an extended study such as in the work of Vitense et al. [46].

Integrating interaction into realistic surgical and interventional settings poses challenges for many researchers. We addressed this aspect by introducing *RQ5*. Upon analyzing the results, it becomes evident that the majority of the MM systems involved evaluation in a simulated scenario rather than a clinical setting, likely because clinical environments were not readily accessible for study purposes. Nonetheless, a close examination of these environments and in-depth workflow analyses, as presented in a work of Schreiter et al. [47], are essential, given that interfaces tested under realistic conditions revealed issues such as low recognition rates of implemented interaction techniques or interference with the medical workflow. In this context, a diligent selection of input modalities is crucial to ensure seamless integration into the workflow. Moreover, the absence of haptic feedback is a prevalent issue in touchless interactions. Current research is engaged in addressing this challenge, and proposed solutions include the usage of ultrasound to project tactile sensations [48]. Additionally, the presence of diverging evaluation methods emphasizes the need for standards, incorporating established questionnaires, and possibly comparison with conventional systems or an unimodal interaction approach. The decision to include non-standardized questionnaires is presumably influenced by a combination of limited awareness of existing ones and the perception that they are less relevant to the indented study objectives. Thus, standardized evaluation is all the more important to facilitate comparability across different systems. In this context, the selection of evaluation metrics should always be adapted to the particular research question(s).

This work contains some limitations. The focused search resulted in a limited amount of high-quality research work. It is therefore advisable to enhance the analysis of MM systems by broadening the scope of the investigation for a comprehensive understanding and broader generalization. This could be achieved by exploring domains that extend beyond the medical context, such the gaming sector or the automotive industry. A cross-disciplinary analysis has the potential to yield valuable insights with regard to the design, usability, and effectiveness of MMI.

## Conclusion

We systematically investigated the advancements of MMI within surgical and interventional scenarios over the past decade. Our analysis addressed specific aspects with regard to the targeted fields and tasks, included input and output modalities, challenges in developing and evaluating such systems, as well as potential future directions in this evolving area of research. Notably, MMI has been frequently implemented for secondary tasks like accessing and navigating medical data, in most cases applying combined voice-hand interaction. The application of gaze, body, and face control has been marginal, likely attributed to ergonomic considerations and difficulties associated with inaccurate tracking of certain body parts. Technical progress facilitates advancements to incorporate MM approaches for HCI. However, particular emphasis should be placed on their development and evaluation. This involves an in-depth analysis of the current workflow, considerations of the surrounding environment, user needs, the selection of number and type(s) of suitable interaction techniques, as well as the application of standardized evaluation methods.

## Data Availability

Not applicable.

## Footnotes

1 (“Multimodal Interaction” OR “Touchless Interaction” OR “Gesture Interaction” OR “Multimodal Interface” OR “Natural User Interface” OR “Touchless Interface” OR “Gesture Interface” OR “Multimodal Control”) AND (“Medical” OR “Medicine” OR “Operating Room” OR “Radiology” OR “Surgery”).

2 Combinations of technical approaches for the same input modality are not considered (i.e., two different hand tracking methods).

3 i.e., laparoscopic instruments and foot switches for virtual training.
